# Impact of the Mediterranean Diet on Athletic Performance, Muscle Strength, Body Composition, and Antioxidant Markers in Both Athletes and Non-Professional Athletes: A Systematic Review of Intervention Trials

**DOI:** 10.3390/nu16203454

**Published:** 2024-10-11

**Authors:** Ellis Bianchi, Hilal Erbasan, Patrizia Riso, Simone Perna

**Affiliations:** Division of Human Nutrition, Department of Food, Environmental and Nutritional Sciences (DeFENS), Università degli Studi di Milano, 20133 Milano, Italy; ellis.bianchi@unimi.it (E.B.); herbasan21@gmail.com (H.E.); simone.perna@unimi.it (S.P.)

**Keywords:** mediterranean diet, sport, athletes, performance, strength, antioxidant markers

## Abstract

**Background:** The Mediterranean Diet (MD) has gained attention for its potential benefits in enhancing athletic performance and overall health. This systematic review aims to evaluate the effects of the MD on athletic performance, strength, body composition, and metabolic markers in both athletes and non-professional athletes. **Methods:** The review included seven studies with a total of 116 participants, ranging from professional handball players to non-professional strength athletes. The studies assessed various aspects of athletic performance, including strength, power, endurance, and body composition. **Results:** The main key findings from the review showed that MD may improve muscle endurance and power, as well as anaerobic performance in CrossFit athletes, and MD was associated with enhanced strength performance, including increased vertical jump height, hand grip strength, and shuttle run performance. The results on the impact on body composition were mixed, with some studies showing improvements in fat-free mass and skeletal muscle mass, while others found no significant changes. The MD also demonstrated positive effects on several markers, such as increased plasma total antioxidant activity and decreased markers of oxidative stress and inflammation. **Conclusions:** In conclusion, while the MD seems to represent a viable dietary strategy for enhancing athletic performance and overall health, more rigorous studies are necessary to clarify its impact across diverse athletic populations.

## 1. Introduction

The Mediterranean Diet (MD) is one of the most well-known and studied dietary patterns [1]. MD is characterized by a high intake of plant-based foods, olive oil, and nuts, and moderate consumption of fish and dairy, also emphasizing the importance of fruits, vegetables, whole grains, and healthy fats like olive oil, all fundamental elements rich in antioxidants substances and polyphenols [2].

Several studies have been conducted over the years on the impact of this dietary pattern, consistently confirming the beneficial effects of greater adherence to the MD in reducing overall mortality and the incidence of major chronic diseases [1,3]. Long-term studies, including the PREDIMED and CORDIPREV trials, have demonstrated the cardiovascular benefits of the MD. Moreover, several randomized controlled trials have shown greater reductions in body weight and BMI, along with improvements in body composition, compared to other diets [4].

Despite the extensive scientific literature documenting the numerous health benefits of the MD, its application within the context of sports nutrition has not been widely explored [5]. The literature available to date shows that adherence to MD is associated with improved power and muscle endurance, as well as favorable body composition in athletes, including its positive impact on cardiovascular health, cognitive function, and overall well-being [6,7,8]. For instance, some studies showed that high adherence to MD is associated with better walking speed and knee muscle strength speed, while others found an improvement in lower limb performance after following a Mediterranean dietary pattern [7,9]. Despite this, relatively few studies have explored the impact of the MD on physical performance, including across various sports disciplines. The available data suggest that it may have a positive effect on several parameters beneficial to the population under study.

The ISSN position stand, along with the position statement from the Academy of Nutrition and Dietetics, Dietitians of Canada, and the American College of Sports Medicine, emphasize the importance of a well-structured diet for physically active individuals, particularly highlighting the critical role of carbohydrates in supporting physical performance. Individuals engaged in general fitness programs can typically meet their nutritional needs with a traditional diet. However, as exercise volume and intensity increase, energy intake may need to reach 40–70 kcal per kg of body weight per day, with a high carbohydrate content to support performance and recovery [10,11]. In the MD pattern, carbohydrates can account for up to 60% of daily energy intake, offering a well-balanced nutrient profile that is suitable for both professional and non-professional athletes, as well as for various types of sports practices, including both endurance and strength athletes [12]. Additionally, the fiber content of a traditional MD can help regulate the glycemic response, mitigating potential glycemic spikes from the high carbohydrate intake required for this population group [13]. While regular physical activity is known to enhance muscle performance and boost energy metabolism, engaging in unfamiliar or excessive exercise can lead to cellular damage. This can compromise muscle function by inducing tissue inflammation and increasing oxidative stress [14]. The growing body of evidence on exercise-induced oxidative damage and its effects on athletic performance has driven significant research in this area [15]. MD is rich in a wide range of important antioxidants and anti-inflammatory compounds, such as bioactives, vitamins, folates, flavonoids, and omega-3 polyunsaturated fatty acids, which have been investigated for their potential muscle-protective effects. Vitamin C, albumin, vitamin E, carotenoids, and flavonoids are able to slow down the oxidation of proteins, lipids, carbohydrates, and DNA and help counteract the oxidative stress induced by physical activity [16,17,18]. Moreover, nutrition plays a crucial role in restoring the immune system after exercise. The existing literature indicates that carbohydrates, along with an appropriate intake of antioxidants and phytochemicals, can attenuate exercise-induced increases in circulating cytokines and the redistribution of neutrophils, monocytes, natural killer cells, and lymphocytes [19].

In this regard, considering its important micronutrients, MD benefits have been attributed to its anti-inflammatory and antioxidant properties, which enhance metabolic efficiency and recovery, particularly for longer and more intense sessions, by supporting sustained energy levels and reducing fatigue [20].

However, overall, its impact on athletic performance, strength, body composition, and metabolic markers in athletes remains an area of active research [6,7,21].

This systematic review aims to comprehensively evaluate the existing literature on the effects of the Mediterranean diet on numerous markers and variables associated with athletic performance in both athletes and non-professional athletes. This work will enhance our understanding of the current scientific state of the art and highlight existing knowledge gaps. By identifying these areas, it will facilitate more comprehensive and focused research on the topic in the future.

## 2. Materials and Methods

This systematic review was conducted in accordance with the Preferred Reporting Items for Systematic Reviews and Meta-Analyses (PRISMA) 2020 PRISMA guidelines [22].

### 2.1. Literature Search and Study Selection

A systematic search was conducted by HE and SP using the following databases: Pubmed, Scopus, and ScienceDirect from 1 June 2024 to 1 August 2024, using the following search strategy and terms: Mediterranean diet [MeSH Terms] AND (athletic performance [MeSH Terms] OR strength [MeSH Terms] OR body composition [MeSH Terms] OR metabolic markers [MeSH, Terms] OR antioxidant markers [MeSH, Terms] OR muscle mass [MeSH, Terms]). An additional manual search was conducted in Google Scholar to identify any relevant studies.

Studies were limited to intervention studies, including clinical trials (randomized or non-randomized), crossover designs, one-arm trials, or pilot studies, all conducted in humans. No date limit was considered in terms of the year of publication, and only studies published in English were considered. Studies were excluded if they did not evaluate the effects of an MD or other dietary interventions on athletes or physically active individuals. Additionally, studies that focused on non-active populations or athletes that were not active at the moment of the evaluations were also excluded.

Two reviewers (HE) and (SP) were tasked with reviewing each article’s title, abstract, and full text. PR and EB did the final checks.

### 2.2. Quality Assessment

Two reviewers (SP and PR) independently evaluated the methodological quality of the eligible studies using the revised Cochrane risk-of-bias tool for randomized trials (RoB-2), which is comprised of five main domains, including bias arising from the randomization process, bias due to deviations from the intended interventions, bias due to missing outcome data, bias in the outcome measurements, and bias related to the selection of reported results. Final opinions and general risk of bias were characterized as “Low” or “High” risk of bias or stated as “Some Concerns”. A third reviewer (EB) checked this assessment.

## 3. Results

**Figure 1:**
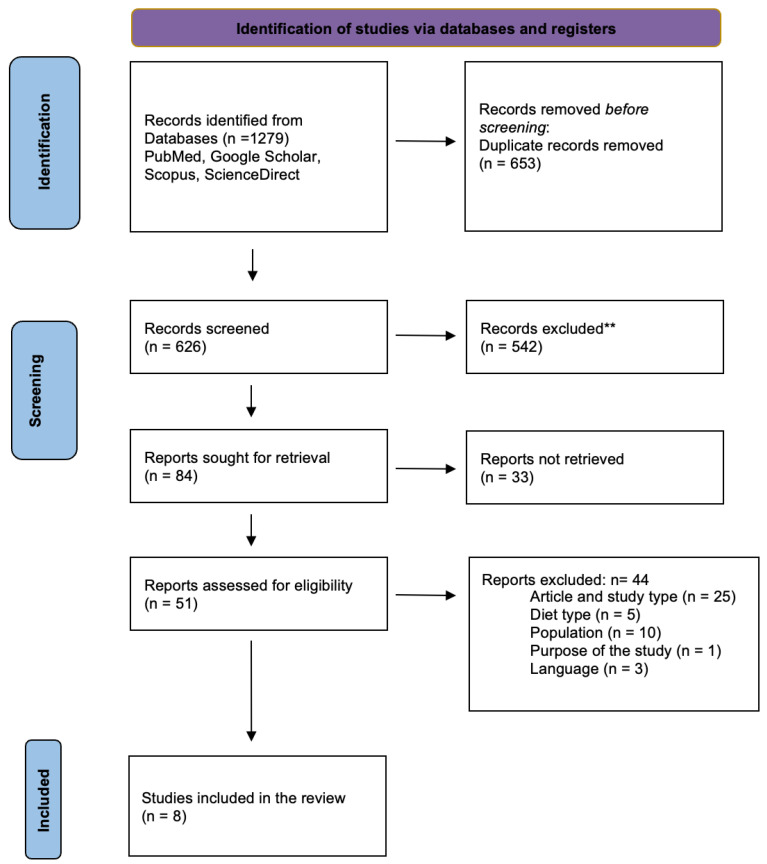
Preferred Reporting Items for Systematic Reviews and Meta-Analyses (PRISMA) flow diagram [22]. ** The records did not meet the eligibility criteria.

A total of eight research articles were eligible to be considered for this systematic review, as shown in the PRISMA diagram in Figure 1.

As shown in Table 1, 1279 records were identified through database searches, including PubMed, Google Scholar, Scopus, and ScienceDirect. After removing duplicates, 626 records were screened based on titles and abstracts. Subsequently, 51 full-text articles were assessed for eligibility, resulting in 8 studies that met the inclusion criteria for this systematic review. The excluded studies varied in design, with 25 cohort studies and cross-sectional studies.

The sample sizes ranged from 11 to 47 participants, with a mean age of 30.0 ± 5.5 years. Most studies focused on athletes and those playing volleyball and ski-running sports. The duration of the interventions varied from 4 days to 8 months. The collective findings from the studies included are summarized in Table 1.

**Figure 2:**
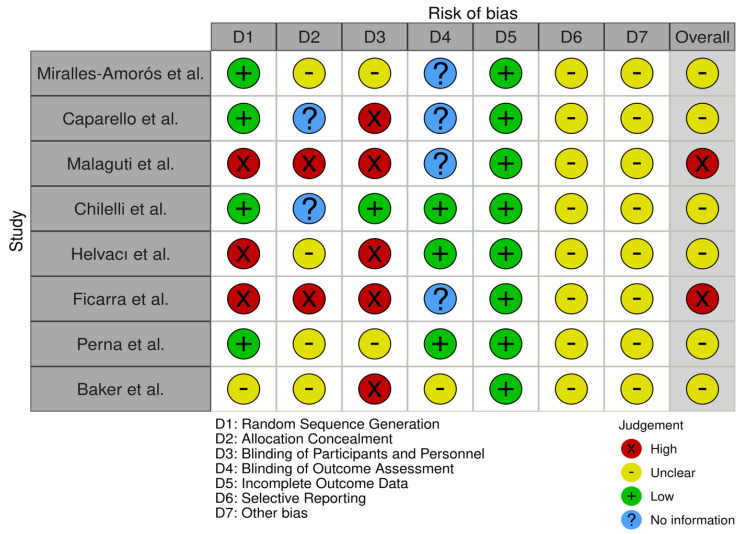
Risk of bias assessment table [23,24,25,26,27,28,29,30].

The risk of bias assessment in the studies was conducted using the revised Cochrane risk of bias tool for randomized trials (RoB 2), and it is reported in Figure 2.

The risk of bias assessment for the included studies was performed independently by HE and EB and revised by SP.

The risk of bias was assessed and found to be moderate in six of the eight studies [23,24,26,27,29,30] and high in two studies [25,28] of the RoB-2 tool (Figure 2).

### 3.1. Athletic and Strength Performance

A study conducted by Ficarra et al. on CrossFit athletes (12 following their traditional diet and 10 following an MD, providing them a personalized diet) reported an increase in peak power (PP) and PP/kg of body weight after the MD or the traditional diet, during the Wingate test. Moreover, max speed significantly increased only in the MD group, and time PP was significantly reduced in the same group. Additionally, the power drop and its related measures were significantly higher or showed a trend toward significance only in the MD group. These results shed light on how an MD could improve anaerobic performance in CrossFit athletes. The same study revealed significant improvements also in strength parameters. Specifically, participants on the MD showed a notable increase in squat jump performance, in particular, jump height increased after 8 weeks of MD. Regarding CrossFit performance, the MD group showed significant improvements in the push-up test to exhaustion, the chin-up test to exhaustion, and the “Fran workout” [28].

Another recent randomized, crossover, controlled trial by Perna et al. has been conducted on 13 non-professional strength athletes. The subjects followed either an MD with a high carbohydrate content (HCMD—55–60% of daily energy derived from carbohydrates) and an MD with reduced carbohydrate content (RCMD—40–45% of daily energy derived from carbohydrates) for 8 weeks, with 6-weeks wash-out period in between. Several tests have been conducted to assess strength performance. No significant differences were found for strength performance parameters except for the elbow flexor maximum voluntary contraction test, indicating a decrease in biceps strength after 8 weeks of RCMD, even when compared to the HCMD intervention [29].

Similarly, Helvacı et al. investigated the impact of a Mediterranean-style diet on strength performance in adolescent ski-running athletes by conducting various tests. Results indicated significant improvements in vertical jump height and hand grip strength, as well as enhanced shuttle run performance, including increased test duration, total distance, and maximum oxygen consumption The study highlighted a reduction in upper middle arm circumference and an increase in height among the athletes [27].

Baker et al. investigated the endurance performance in a randomized-sequence crossover study after 4 days of MD and Western diet. Following the MD, participants completed a 5 km treadmill time trial an average of 6% ± 3% faster, equating to a reduction of 1.5 ± 0.6 min compared to the Western diet group. Notably, 10 out of 11 participants achieved quicker run times when adhering to the MD. Additionally, the average running speed during the third kilometer was higher in the MD group, further highlighting the performance benefits of the Western diet. No significant differences were found in different parameters related to anaerobic endurance performance during the Wingate Anaerobic test. Similar results were also found for hand grip strength and vertical jump performance [30].

The study conducted by Miralles-Amorós et al. that involved 21 professional female handball players and evaluated the effects of personalized dietary plans, including a (MD), on physiological and physical variables related to Relative Energy Deficiency in Sport (RED-S) over a 12-week randomized controlled trial. Regarding strength performance, significant differences were observed only in the Abalakov jump test, with jump height showing improvement over the study period [23].

### 3.2. Body Composition

The study conducted by Perna et al. noticed a significant decrease in arm circumference following the intervention with reduced carbohydrate content, with no significant difference when compared to the higher carbohydrate intervention. A similar result has been observed also for waist circumference. Bicipital skinfold and body fat percentage significantly decreased after the HCMD compared to baseline, while no changes were observed following the RCMD. Anthropometric measurements were taken by a physician with extensive experience in anthropometry, using a Holtain Tanner/Whitehouse Skinfold Calliper to measure the skinfold thickness [29].

In the study conducted by Malaguti et al., no statistically significant results were observed in terms of BMI and body fat percentage changes after 2 months following an MD [25]. Similar results have been found by Ficarra et al., where no significant changes were observed in anthropometric measurements or body composition parameters after the 8-week intervention [28]. The two aforementioned studies used different methods to assess body composition: Malaguti et al. utilized skinfold measurements, while Ficarra et al. applied bioelectrical impedance analysis in an upright position. Both methods were able to estimate specific parameters, such as body fat percentage. Likewise, in the study conducted by Helvacı et al., no statistically significant differences in key body composition metrics such as body fat percentage, lean body mass, and total body water percentage before and after the 15-day intervention [27]. The study conducted by Caparello et al. observed significant improvements in body composition among volleyball athletes following the MD during the Italian Championship. Specifically, a significant increase in fat-free mass (FFM) was noted post-intervention, indicating an enhancement in muscle mass. Furthermore, skeletal muscle mass (SMM) showed a marked increase, reflecting improved muscle development [24]. All the parameters mentioned in the two recent studies were estimated using the bioelectrical impedance method.

In the study conducted by Miralles-Amorós et al., significant changes in certain body composition parameters were detected over time, while differences between groups were only observed in fat-free mass, lean mass, and total body water. All the analysis was conducted by the same investigator, an ISAK level 2 anthropometrist, using skinfold measurements and the dual indirect bioelectrical impedance method [23]. Circumferences were consistently measured using a non-elastic tape.

### 3.3. Metabolic and Antioxidant Markers

Perna et al. showed a significant increase in MCV, and a decrease in MPV were observed following HCMD intervention, although these changes fell within the normal physiological range. In contrast, RCMD did not result in any statistically significant changes over time [29].

In the study conducted by Malaguti et al., plasma total antioxidant activity (TAA) was measured in both groups (MD group and a high-protein, low-calorie group supplemented with omega-3), showing a significant increase after the 2 months of MD, indicating enhanced antioxidant defenses [25].

In the study conducted by Chilelli et al., the MD group experienced significant decreases in sRAGE (soluble receptor for AGE), NEFA (non-esterified fatty acids), and MDA (malondialdehyde) compared to the baseline. The plasma phospholipid fatty acid (PPFA) composition remained unchanged between the two groups [26].

## 4. Discussion

This systematic review comprehensively evaluates the impact of the Mediterranean diet (MD) on athletic performance, strength, body composition, and physiological/metabolic markers in both amateur and professional athletes. The findings reveal a complex yet promising relationship between adherence to the MD and various outcomes, suggesting that this dietary pattern could be a valuable tool for optimizing both athletic performance and strength in athletes, though results vary across different studies and interventions.

The review revealed mixed results regarding the impact of the MD on athletic and strength performance. Specifically, the included studies suggest that the MD can positively influence both strength and athletic performance, particularly with respect to anaerobic performance.

The importance of a balanced diet, with an appropriate amount of carbohydrates, for supporting athletic and strength performance in athletes is well known [10,11]. Current research shows that athletes and sports subjects often fail to meet the optimal intake of energy and carbohydrates while exceeding recommendations for fat and protein [31]. Some evidence suggests that carbohydrate restriction may impair anaerobic and strength performance [32,33]. Due to these concerns, a balanced MD with the appropriate proportion of macronutrients, particularly an optimal amount of carbohydrates, could be an ideal dietary pattern for this population group. In fact, a traditional MD is considered balanced and well-rounded, containing carbohydrates ranging from 45% to 60% of the total daily energy intake [12].

Moreover, the Mediterranean dietary pattern is considered an excellent source of various nutrients due to the consumption of a wide variety of foods, including extra virgin olive oil, cereals, nuts, legumes, fruits, and vegetables. It is particularly rich in antioxidants such as vitamin E, β-carotene, vitamin C, and flavonoids, as well as minerals like selenium and folates [34]. All these components are considered highly important, as they can protect against oxidative stress and inflammation. This could be particularly beneficial for athletes, helping to counteract the stress induced by physical exercise. One possible mechanism could be the enhancement of endothelium-mediated vasodilation and muscle perfusion, ultimately contributing to improved motor performance [35].

Concerning anthropometric characteristics and body composition, most studies reported no significant differences before and after an MD intervention, except for a few that showed significant improvements in certain parameters (e.g., fat-free mass). First, it should be noted that the individuals involved in these studies, whether amateur or professional athletes, generally already had an optimal body composition within the normal range. Therefore, a radical change would not be expected, even with the adoption of a balanced dietary pattern. It is well-known that a balanced and well-structured dietary pattern is the most important thing to meet the needs of athletes and to maintain body composition and anthropometric parameters within normal ranges [36]. Moreover, the MD is considered optimal for calcium bioavailability, and this could explain the slight improvement in fat-free mass and skeletal muscle mass found in the study conducted by Caparello et al. [24,37]. Overall, the MD has the potential to enhance and maintain body composition by offering the necessary nutritional support for sustaining high-level performance while also providing a low-inflammatory diet rich in antioxidants, essential vitamins, and minerals. For this reason, it is considered a dietary pattern with low micronutrient deficiency risk [6]. Overall, it must be taken into consideration that the study used different methodologies, showing a certain heterogeneity, for assessing body composition parameters. In particular, skinfold measurements and bioimpedance analysis were used in certain studies to estimate body fat percentage and other parameters. It is important to note that anthropometry is a method characterized by high variability between operators and requires the use of formulas to estimate body fat mass and other parameters. A similar concern applies to bioelectrical impedance analysis, which can only estimate certain body composition parameters based on body water content [38].

The impact of the MD on metabolic and antioxidant markers was heterogeneous, with various studies analyzing different parameters and showing a general trend toward improvement in several health-related markers. The study conducted by Malaguti et al. noticed a significant improvement in total antioxidant activity (TAA) after the MD intervention [25]. Another selected study evaluated the effect of an MD on the Glyco-Oxidative Status and Lipo-Oxidation in master athletes, noticing an improvement in different health-related parameters analyzed [26]. It is well-known that physical activity leads to an increase in the production of reactive oxygen species. However, it has also been recognized that physical activity can promote the induction of antioxidant enzymes, thereby enhancing antioxidant defenses [39]. Similarly, physical exercise performed at high intensity and volume can elevate free radical and ROS levels, potentially impeding recovery by overwhelming the body’s natural antioxidant defenses [40]. Consuming foods rich in antioxidants and bioactive compounds, as found in a Mediterranean dietary pattern, may help accelerate the recovery process in athletes and sports subjects [34,41]. Furthermore, it is now well-established that an MD can improve various health-related parameters, including lipid profiles, and can help in managing the intake of advanced glycation end products (AGEs) [42,43,44]. A food traditionally present in the Mediterranean dietary pattern is extra-virgin olive oil, which is considered an excellent source of fatty acids and other important nutrients. Due to its high polyphenol content, including compounds such as hydroxytyrosol and oleuropein, it exhibits antioxidant properties [45]. Additionally, extra-virgin olive oil is rich in monounsaturated fatty acids, particularly oleic acid, which is well-known for its health benefits [46]. All these concerns are consistent with those observed in the study conducted by Perna et al., which found that an MD is considered an optimal dietary pattern for maintaining blood parameters within normal ranges in amateur strength athletes [29].

### Limitations and Future Research

While this review provides valuable insights into the effects of the MD on athletic performance, strength, body composition, and metabolic markers, several limitations should be acknowledged. First, the studies included in this review varied in terms of study design, population, and intervention duration, which may have contributed to the heterogeneity of the results. Additionally, the small sample sizes and short intervention periods in some studies may limit the generalizability of the findings.

Future research should aim to address these limitations by conducting larger, well-designed, randomized controlled trials with longer intervention periods. Moreover, studies should explore the specific components of the MD that contribute to its beneficial effects, as well as the potential interactions between the MD and other dietary supplements or training regimens. Investigating the impact of the MD on different athlete populations, including those with different training levels and sports disciplines, would also provide valuable insights into the diet’s applicability across various contexts. Moreover, from a practical perspective, it will be compelling in the future to evaluate also the impact and feasibility of a Mediterranean dietary pattern during different phases of training and recovery during the season to adapt the dietary pattern to the different phases of the training cycle during the year, especially for professional athletes.

## 5. Conclusions

In conclusion, the Mediterranean diet shows promise as a dietary strategy for improving athletic performance, strength, and body composition in athletes, with potential benefits for metabolic health. However, the variability in study results underscores the need for further research to fully elucidate the mechanisms underlying these effects and to determine the most effective ways to implement the MD in sports nutrition. By integrating the MD into training and recovery protocols, athletes, coaches, and sports nutritionists may be able to optimize performance and overall well-being, contributing to long-term athletic success.

## Figures and Tables

**Table 1:** Characteristics of the selected studies (n = 8), including population, treatment, study design and duration, and key findings from each.

First Author/Year of Publication	Population	Treatment	Study Design and Duration	Main Results
Miralles-Amorós et al./2023. [23]	21 female handball players (Aged 22 ± 4 years)	Divided into three intervention groups: Free diet (n = 7) Mediterranean diet (n = 7) Antioxidant Diet (n = 7)	Parallel randomized control study 12 weeks	No significant differences were found in terms of health-related markers (e.g., cholesterol). Significant differences were found for some body composition parameters, but only over time; significant differences between groups were observed only in fat-free mass, lean mass, and total water. In terms of strength performance, significant differences were found over time only for the Abalakov jump test, with jump height improving over time.
Caparello et al./2023 [24]	11 Volleyball male players (Aged 27 ± 6 years old)	Mediterranean diet (n = 11)	Longitudinal study 8 months	Significant differences were found for body composition and bioelectrical parameters after an optimal adherence to MD. Specifically, a statistically significant improvement was found in all parameters, except for those related to hydration status.
Malaguti et al./2008 [25]	11 nonprofessional male volleyball athletes (Group A aged 28.8 ± 4.7 years, Group B aged 31.7 ± 4.1 years)	Group A: Mediterranean diet (n = 5) Group B: High-protein, low-calorie diet (n = 6)	Parallel study 2 months	No significant differences were found after 2 months of MD for body composition parameters. Plasma total antioxidant activity (TAA) increased significantly after 2 months of MD, indicating that physical activity, rather than the differing diets, is the primary factor contributing to the rise in plasma TAA. However, the study did not find any significant changes in the fatty acid composition of red blood cell membranes after the supplementation period.
Chilelli et al./2016 [26]	47 healthy male athletes (Aged 46 ± 8 years)	Group 1 Mediterranean diet alone (MD group n = 22) Group 2 MD plus curcumin and BSE (curcumin/BSE group n = 25)	Parallel randomized control study 3 months	No significant differences were found for body composition parameters after both intervention periods. MD with an addition of curcumin/BSE had a positive effect on glycoxidation and lipid peroxidation. Compared to MD alone group curcumin/BSE group showed a significant decline in total advanced glycation end products. Both groups had a significant decrease in soluble receptor for AGE (sRAGE), non-esterified fatty acids (NEFA), and malondialdehyde (MDA); the changes in total AGE and MDA differed significantly between the two groups. Neither group exhibited significant changes in inflammatory markers after the intervention, except for a slight increase in TNFα in the MD group.
Helvacı et al./2023 [27]	15 professional male ski-running sports athletes (Aged 14.9 ± 1.3 years)	Mediterranean diet (n = 15)	Longitudinal study 15 days	No significant differences were observed for body composition parameters before and after the intervention period. MD intervention improved exercise performance and reduced perceived fatigue without significantly altering lactate elimination or body composition. MD diet increased vertical jump height, hand grip strength, and parameters of the 20 m shuttle run test). Perceived fatigue scores decreased at the several stages of the shuttle run test.
Ficarra et al./2022 [28]	13 men and 9 women (Diet group aged 38.3 ± 8.9 years, Control group (Aged 35.6 ± 8.4 years)	A diet group n = 10 (DG/Mediterranean diet plus CrossFit training) A control group n = 12 (CG/Habitual diet plus CrossFit training)	Parallel study 8 weeks	No significant differences were found for body composition parameters before and after each intervention period in both groups. In the DG group, but not in the CG, there were improvements in circumference measures and significant improvement in squat jump performance, power, muscular endurance, and anaerobic capacity. Furthermore, after 8 weeks on the MD, participants showed enhanced CrossFit-specific performance.
Perna et al./2024 [29]	9 males and 4 females (Mean age 25.8 ± 4.2)	Interventions: High Carbohydrate Mediterranean Diet (HCMD—55–60% of carbohydrates) and Reduced Carbohydrate Mediterranean Diet (RCMD—40–45% of carbohydrates)	Crossover randomized control study 8 weeks + 6 weeks washout + 8 weeks	No significant differences were found for almost all strength and body composition parameters, except for biceps strength, which showed an increase in performance after 8 weeks of HCMD, even when compared to the RCMD treatment. This improvement was paired with a reduction in biceps circumference, which was seen exclusively after the RCMD. Regarding the impact on blood markers, significant differences were observed, but all remained within the normal range.
Baker et al./2019 [30]	4 males and 7 females (Aged 28 ± 3 years)	Group 1: Mediterranean Diet Group 2: Western diet	Randomized-sequence crossover study 4 days + 4 days	Five-kilometer run time was 6% ± 3% shorter in the MD group than in the Western diet group. No significant differences were found between the diet conditions for anaerobic exercise tests, including peak and mean power from the Wingate test, as well as performance in the vertical jump test and hand grip strength test.

## Data Availability

All the information used is available to the public. The data extracted from the studies and any other material used in the review are presented in the different sections of the work or referenced in the bibliography.
